# Supplementary figures and images for: Improving the workflow to crack Small, Unbalanced, Noisy, but Genuine (SUNG) datasets in bioacoustics: The case of bonobo calls
Source: PLoS Comput Biol. 2023 Apr 13;19(4):e1010325. doi: 10.1371/journal.pcbi.1010325 (PMC10129004; doi:10.1371/journal.pcbi.1010325)

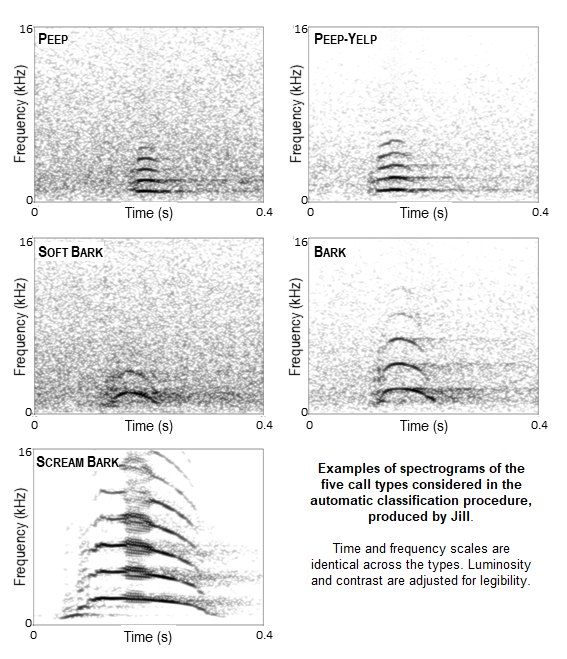

Supplement: S1 Fig — (TIFF) [file pcbi.1010325.s002.tiff]
