# Supplementary material for: Improving the workflow to crack Small, Unbalanced, Noisy, but Genuine (SUNG) datasets in bioacoustics: The case of bonobo calls
Source: PLoS Comput Biol. 2023 Apr 13;19(4):e1010325. doi: 10.1371/journal.pcbi.1010325 (PMC10129004; doi:10.1371/journal.pcbi.1010325)
Supplement: S1 Table — (PDF) [file pcbi.1010325.s003.pdf]

Supplementary Information: Description of the acoustic features and feature sets

In the table below, the first two columns contain the code and description of the various acoustic features. The third column details the composition of the feature sets. A tick indicates that the feature was included in the DFA analyses (see Methodology DFA for details).

Code	Description	Sets
duration	Call duration (s)	Bioacoustics✓<br>DCT✓
vocalization.HNR	Harmonics-to-noise ratio (dB)	Bioacoustics✓<br>DCT✓
q1f	First quartile of the call energy along the frequency dimension (Hz)	Bioacoustics✓
q2f	Median of the call energy along the frequency dimension (Hz)	Bioacoustics
q3f	Third quartile of the call energy along the frequency dimension (Hz)	Bioacoustics✓
f.max	Maximum fundamental frequency reached over the call (Hz)	Bioacoustics✓
q1t	First quartile of the call energy along the relative time dimension (%)	Bioacoustics✓
q2t	Median of the call energy along the relative time dimension (%)	Bioacoustics
q3t	Third quartile of the call energy along the relative time dimension (%)	Bioacoustics✓
t.max	relative time at which f.max is reached (%)	Bioacoustics
f0.sta	Fundamental frequency at the beginning of the call (Hz)	Bioacoustics
f0.mid	Fundamental frequency at the temporal middle of the call (Hz)	Bioacoustics
f0.end	Fundamental frequency at the end of the call (Hz)	Bioacoustics✓
f0.av	Average fundamental frequency over the call (Hz)	Bioacoustics
f0.slope.asc	Fundamental frequency slope (start-maximum), normalized by time	Bioacoustics✓
f0.slope.desc	Fundamental frequency slope (maximum-end), normalized by time	Bioacoustics✓
f0.slope.1st.half	Fundamental frequency slope (start-middle), normalized by time	Bioacoustics
f0.slope.2nd.half	Fundamental frequency slope (middle-end), normalized by time	Bioacoustics
f0.onset	Raw fundamental frequency slope (start-middle), in Hertz	Bioacoustics
f0.offset	Raw fundamental frequency slope (middle-end), in Hertz	Bioacoustics
dct0	DCT coefficient 0	DCT✓
dct1	DCT coefficient 1	DCT✓
dct2	DCT coefficient 2	DCT✓
dct3	DCT coefficient 3	DCT✓
dct4	DCT coefficient 4	DCT✓
MFCC[1-32]_mean	Average value of the Mel-frequency cepstral coefficients (MFCCs) over the call (unitless)	MFCC✓

ΔMFCC[1-32]_mean	Average value of the ΔMFCCs over the call (unitless)	MFCC✓
ΔΔMFCC[1-32]_mean	Average value of the ΔΔMFCCs over the call (unitless)	MFCC✓
MFCC[1-32]_std	Std Deviation of the MFCCs over the call (unitless)	MFCC
ΔMFCC[1-32]_std	Std Deviation of the ΔMFCCs over the call (unitless)	MFCC
ΔΔMFCC[1-32]_std	Std Deviation of the ΔΔMFCCs over the call (unitless)	MFCC
