# Supplementary material for: Improving the workflow to crack Small, Unbalanced, Noisy, but Genuine (SUNG) datasets in bioacoustics: The case of bonobo calls
Source: PLoS Comput Biol. 2023 Apr 13;19(4):e1010325. doi: 10.1371/journal.pcbi.1010325 (PMC10129004; doi:10.1371/journal.pcbi.1010325)
Supplement: S1 Text — (PDF) [file pcbi.1010325.s004.pdf]

Supplementary Information: Comparison of the classification performances of SVM with different MFCC sets

We assess here how different options for MFCC parameterization impact classification performances on both call types and individual signatures. For the sake of simplicity, we consider a single classifier – SVM – and a single measure of performance – balanced accuracy.

In complement to the original MFCC set considered in the main paper and which consist in are average values for time series of MFCC, ΔMFCC (the first order derivatives of MFCC) and ΔΔMFCC (the second order derivatives of MFCC), we created three reduced sets:

1. **MFCC-reduced,** which contains 32 MFCC computed at the middle of the call only
2. **MFCC-reduced-delta,** which contains 32 MFCC and 32 ΔMFCC computed at the middle of the call
3. **MFCC-reduced-delta-delta** which contains 32 MFCC, 32 ΔMFCC, and 32 ΔΔMFCC computed at the middle of the call

Hyper-parameter tuning for SVM was conducted for each set, as is done in the article.

Regarding the classification of call types, all reduced feature sets lead to clearly weaker performances than those reported in the main paper (see Table S1). One can, however, notice the positive impact on the performances of adding the first and second order derivatives.

Table S1. Classification performances on call types, assessed with balanced accuracy, for the three reduced sets of MFCC and the original set reported in the main paper.

Acoustic Feature Sets	Balanced accuracy
**Original MFCC set (main p**aper)	**0.672**
**MFCC-reduced-delta-delta**	0.614
**MFCC-reduced-delta**	0.583
**MFCC-reduced**	0.523

As for the classification of individual signatures, the pattern of results is similar to the one for call types: taking derivatives into account increases performances but calculating the 32 MFCC coefficients only in the middle of each call results in worse performances compared to the original MFCC set reported in the main paper.

Table 2. Classification performances on individual signatures, assessed with balanced accuracy, for the three reduced sets of MFCC and the original set reported in the main paper.

Acoustic Feature Sets	Balanced accuracy
Original MFCC set (main paper)	**0.447**
**MFCC-reduced-delta-delta**	0.351
**MFCC-reduced-delta**	0.348
**MFCC-reduced**	0.309
