# Supplementary material for: Improving the workflow to crack Small, Unbalanced, Noisy, but Genuine (SUNG) datasets in bioacoustics: The case of bonobo calls
Source: PLoS Comput Biol. 2023 Apr 13;19(4):e1010325. doi: 10.1371/journal.pcbi.1010325 (PMC10129004; doi:10.1371/journal.pcbi.1010325)
Supplement: S2 Text — (PDF) [file pcbi.1010325.s005.pdf]

Supplementary Information: Controlling for call types in the individual signature task

We report here the performances reached with the approach aimed at limiting information leakage described in the article in III. Addressing possible data leakage / Leakage control: some limits with a SUNG dataset.

As a reminder, we work withed a reduced dataset aimed at increasing the minimum number of observations for each pair of individual and call type. Only 5 individuals and 4 types remain, for a total of 902 observations:

	B	P	PY	SB	SCB
Bolombo	6	27	24	17	2
Busira	11	18	34	5	3
Djanoa	20	18	49	36	22
Hortense	9	26	50	8	18
Jill	50	24	89	112	87
Kumbuka	23	35	26	25	0
Lina	8	24	18	23	3
Vifijo	12	20	61	51	16
Zamba	21	43	56	55	18
Zuani	114	20	36	50	37

➔

	B	P	PY	SB
Djanoa	20	18	49	36
Jill	50	24	89	112
Kumbuka	23	35	26	25
Zamba	21	43	56	55
Zuani	114	20	36	50

We considered the three classifiers **svm**, **nn** and **xgboost**, and the three target sets of predictors **Bioacoustic**, **DCT** and **MFCC**. The classification task pertains to individual signature, and the issue we investigate relates to the non-independence of calls due to call types, in other words the possibility that call types display idiosyncratic variation at the individual level.

Following the partition strategies explained in the article and exemplified in Figure 14, we generated all the possible configurations for the training and test sets with for each individual three call types in the training set and one call-type in the test set. That is a total of 4^5 = 1,024 configurations, each with its specific sizes for the training and test sets. We measured the classification performance in each configuration with balanced accuracy, as well as the performance in a matched configuration where for each individual we shuffled the distribution of its calls across the training and test sets – preserving the size of the two sets but introducing leakage. This procedure created a baseline for comparison and proper assessment of the leakage impact. Results for Individual identification are reported in Fig. 1 below.

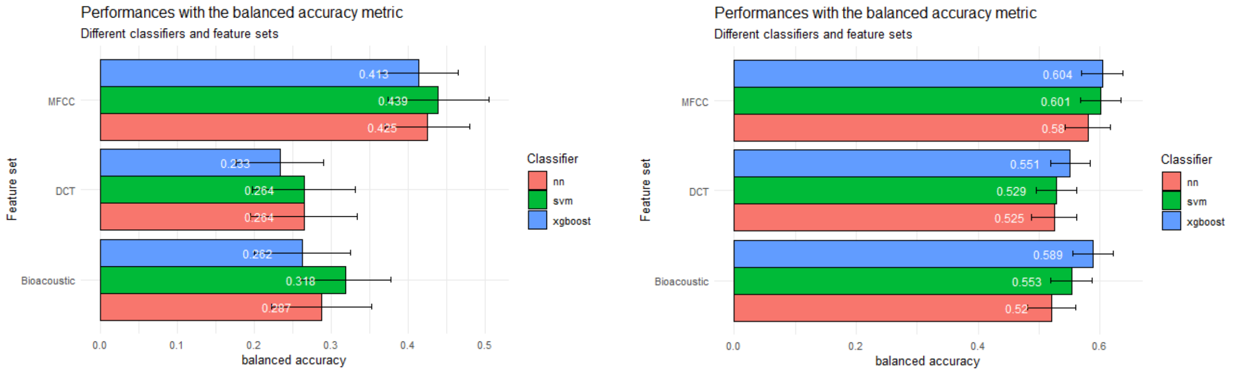

Figure 1. Comparisons of the performances of different classifiers for different feature sets with (left) and without (right) control for call types when predicting the individuals. The error bars represent the standard deviation of the performances across the 1,024 configurations.

As could be expected, we observe that the performances are degraded when controlling for interaction. We also see from the error bars that they vary quite a lot across the 1,024 configurations. This suggests that with such a reduced dataset, each specific repartition of calls between the training and test sets has a significant impact on the model performance. Additionally, while **xgboost** was consistently better in our princeps study and here in the 'uncontrolled' situation, this is no longer the case for reasons that would need a more systematic investigation to be identified.

It appears that the sizes of the training and test sets resulting from the block shuffling vary a lot, with around 60% of variation between the smallest and the largest training sets. We looked at the relationship between the size of the training set and the level of performance in search of a possible weighting scheme to neutralize the effect of the former. Across the different classifiers and sets of predictors, we found a consistent pattern displayed in Fig. 2, where the larger the training set, the worse the balanced accuracy:

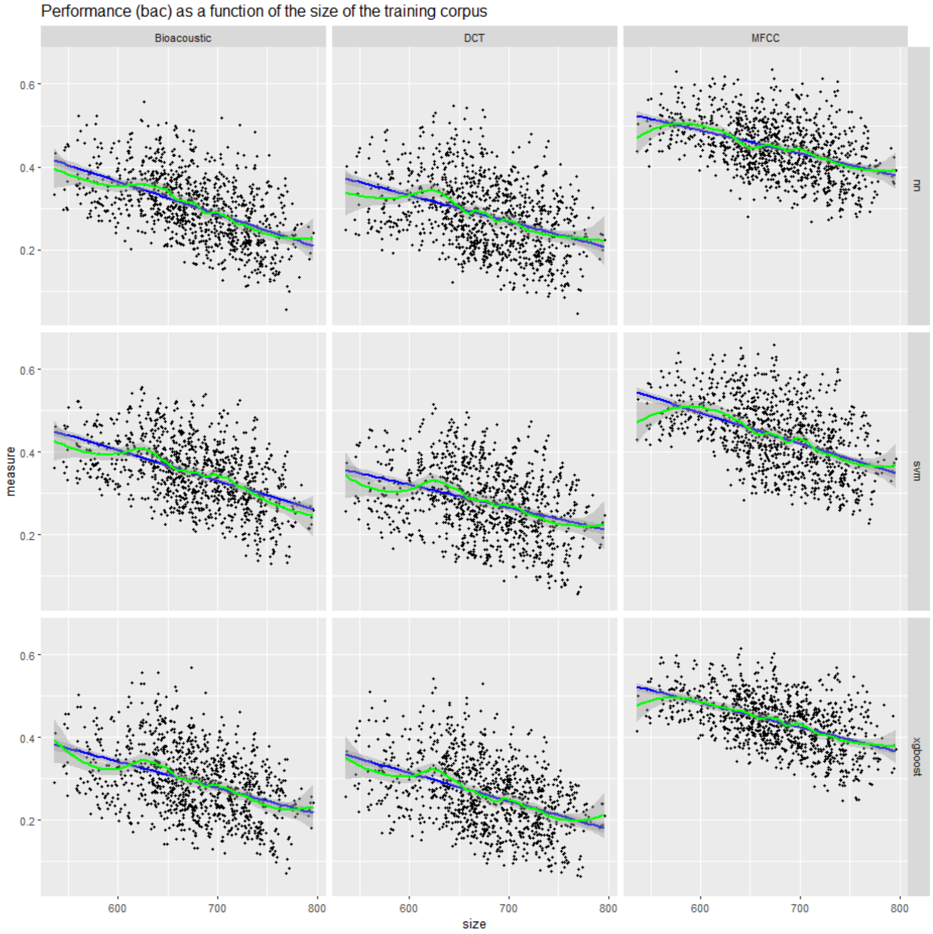

Figure 2. Performance (balanced accuracy) as a function of the size of the training set for different classifiers and feature sets when controlling for call types. The blue line corresponds to a linear regression, while the green one corresponds to a Loess regression.

The previous pattern is rather counter-intuitive, as more data in the training set should intuitively lead to better performances. This suggests that the individual-specific distribution of calls across types is a confounding variable here. To examine this possibility further, we applied a penalized

linear regression (either lasso or ridge) to predict the performance with predictors such as the size of the training and test sets and the ratio of B, P, PY or SB calls in the training set:

Table 1. Coefficients for the various predictors of a penalized regression (either lasso or ridge) with as predicted variable the balanced accuracy of the classification with the test set (1,024 configurations for the training and test sets)

**Predictor**	**Value of the coefficient in the lasso regression model**	**Value of the coefficient in the ridge regression model**
Ratio of B calls in the training set	-0.13	0.0
Ratio of P calls in the training set	0.67	0.84
Ratio of PY calls in the training set	0.19	0.33
Ratio of SB calls in the training set	-0.26	-0.13
Size of the training set	< 0.001	< 0.001
Size of the test set	< 0.001	0.0

The results reported in Table 1 do not offer a comprehensive answer, but they suggest that performance varies quite strongly depending on which call types, from which individual, are in the training and test sets overall. This is likely the result of a combination of several factors: how much information there is in each call type, the imbalance in our dataset, and the graded nature of the bonobo repertoire of calls.
