# Supplementary material for: Improving the workflow to crack Small, Unbalanced, Noisy, but Genuine (SUNG) datasets in bioacoustics: The case of bonobo calls
Source: PLoS Comput Biol. 2023 Apr 13;19(4):e1010325. doi: 10.1371/journal.pcbi.1010325 (PMC10129004; doi:10.1371/journal.pcbi.1010325)
Supplement: S3 Text — (PDF) [file pcbi.1010325.s006.pdf]

Supplementary Information: Illustration of the difference between linear and nonlinear decision boundaries

In our article, we demonstrate that DFA underperforms compared to other classification techniques such as SVM, neural networks and xgboost. A likely explanation lies in the different decision boundaries that these various techniques come up with when learning to classify observations. It is uneasy to explore such decision boundaries with a large number of predictors, i.e., a decision space with a large number of dimensions. We can, however, illustrate the differences between classifiers with an exploratory and pedagogical example where only two predictors / dimensions are considered.

We consider the classification of call types, for which the DCT set of parameters (*duration*, *vocalization.HNR*, *dct0*, *dct1*, *dct2*, *dct3* and *dct4*) leads to good performances, with *duration* and *dct2* playing an important role in the predictions.

To shift from the seven predictors of the full set to two predictors with good discriminatory power, we perform a principal component analysis (PCA) on the basis of our 1,560 calls and consider only the first two principal components (PC).

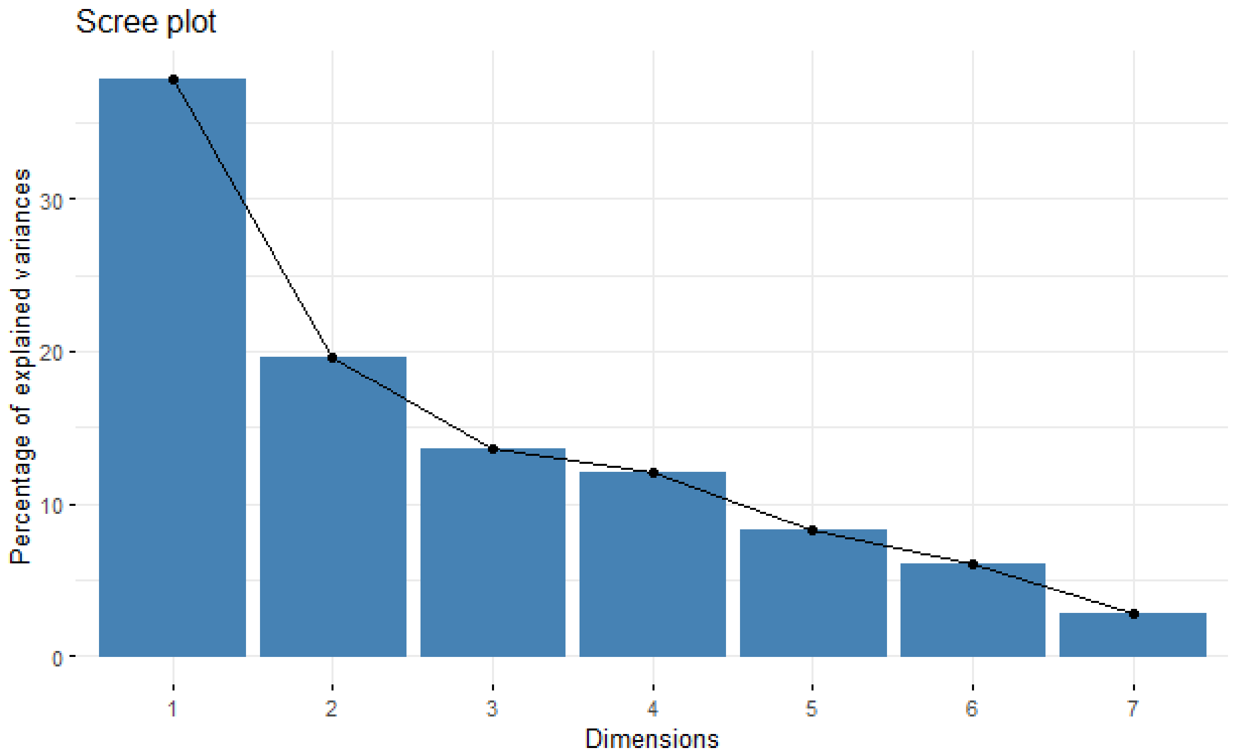

Figure 1. Scree plot for the PCA applied to the 1,560 calls described with the DCT set of parameters.

As shown in Fig. 1, the first two PCs account for respectively 37.8% and 19.6% of the total variance, hence 57.4% together. As shown in Fig. 2, PC1 strongly correlate with *duration*, *dct2* and *dct4*, while PC2 strongly correlate with *dct1*.

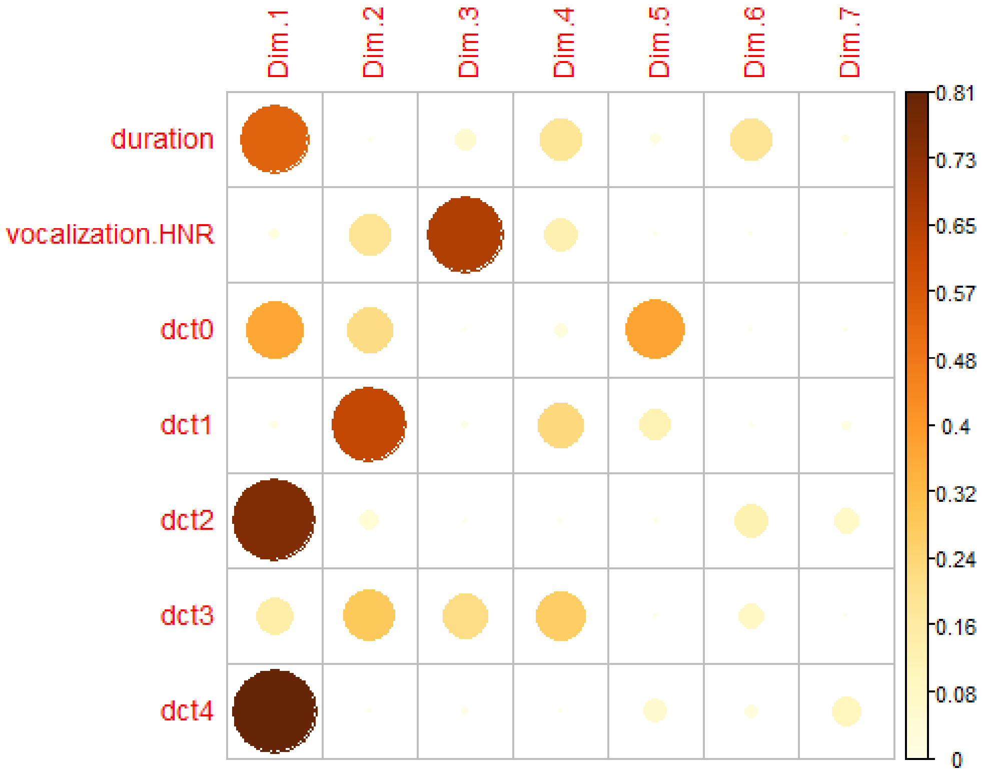

Figure 2. Correlation between the PCs and the initial predictors.

Given the coordinates of our 1,560 calls along the two main dimensions of the PCA, we then rely on the ***plotLearnerPrediction()*** function of the *mlr* package in R to visualize decision boundaries and classification results for 3 classifiers: DFA, svm and xgboost. The function indeed returns a plot which displays these elements after training and assessing the classifier (with a random 75%-25% split for the training and test sets, and a 10-fold cross-validation process to better assess the performance on the test set).

On Fig. 3, Fig. 4 and Fig. 5, one can clearly see the differences between the decision boundaries of the different classifiers. While for DFA, these boundaries appear as made of one or several segments, those of SVM are clearly non-linear. Decision areas for xgboost consist of many rectangular areas, which reflect the fact that the approach is based on decision trees which branches are defined by decision thresholds for the values of the predictors. The overall less complex boundaries for DFA explain the lower classification performances.

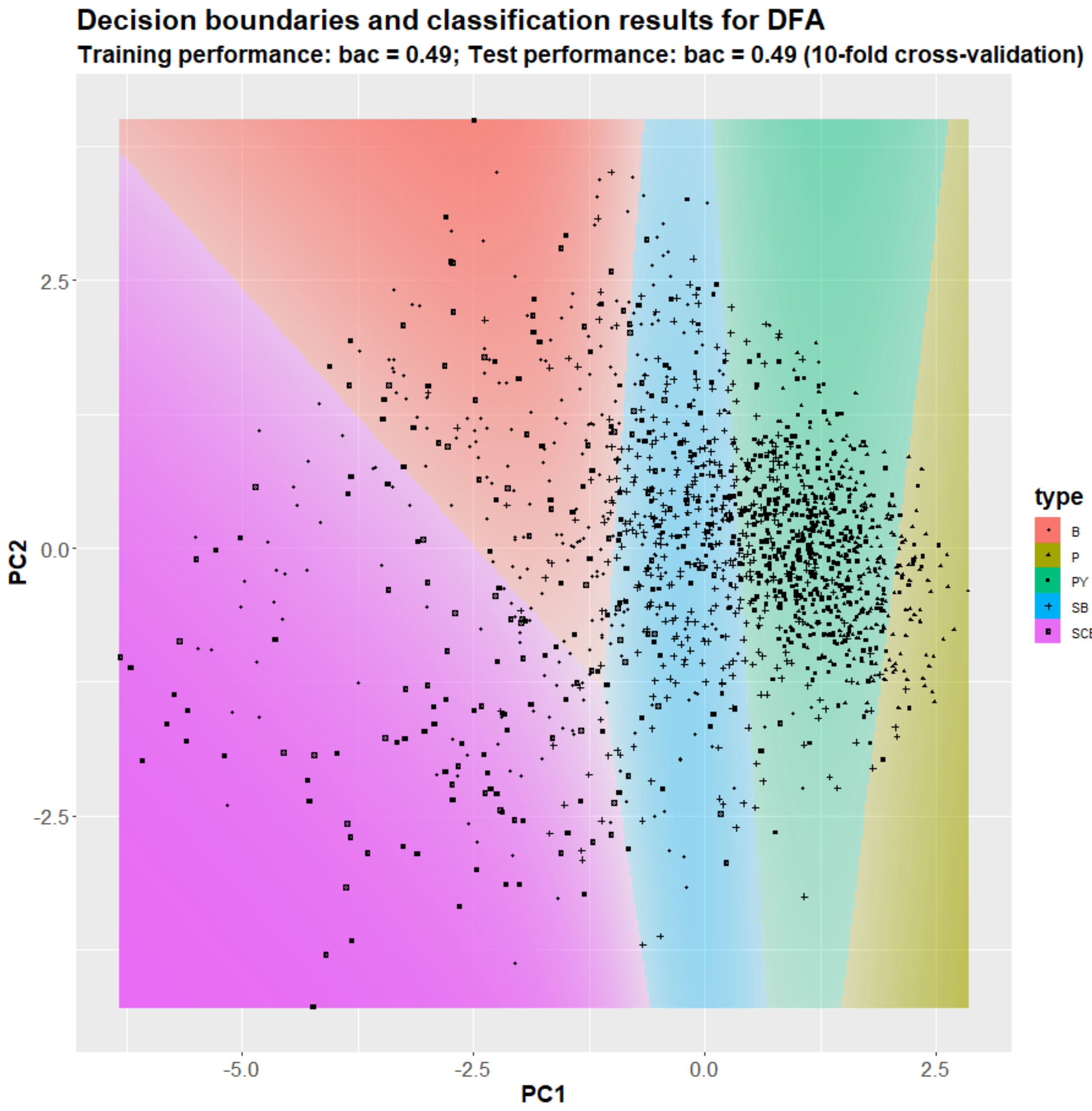

Figure 3. Decision boundaries and classification results for DFA. Darker colors for the decision areas correspond to higher confidence in the prediction.

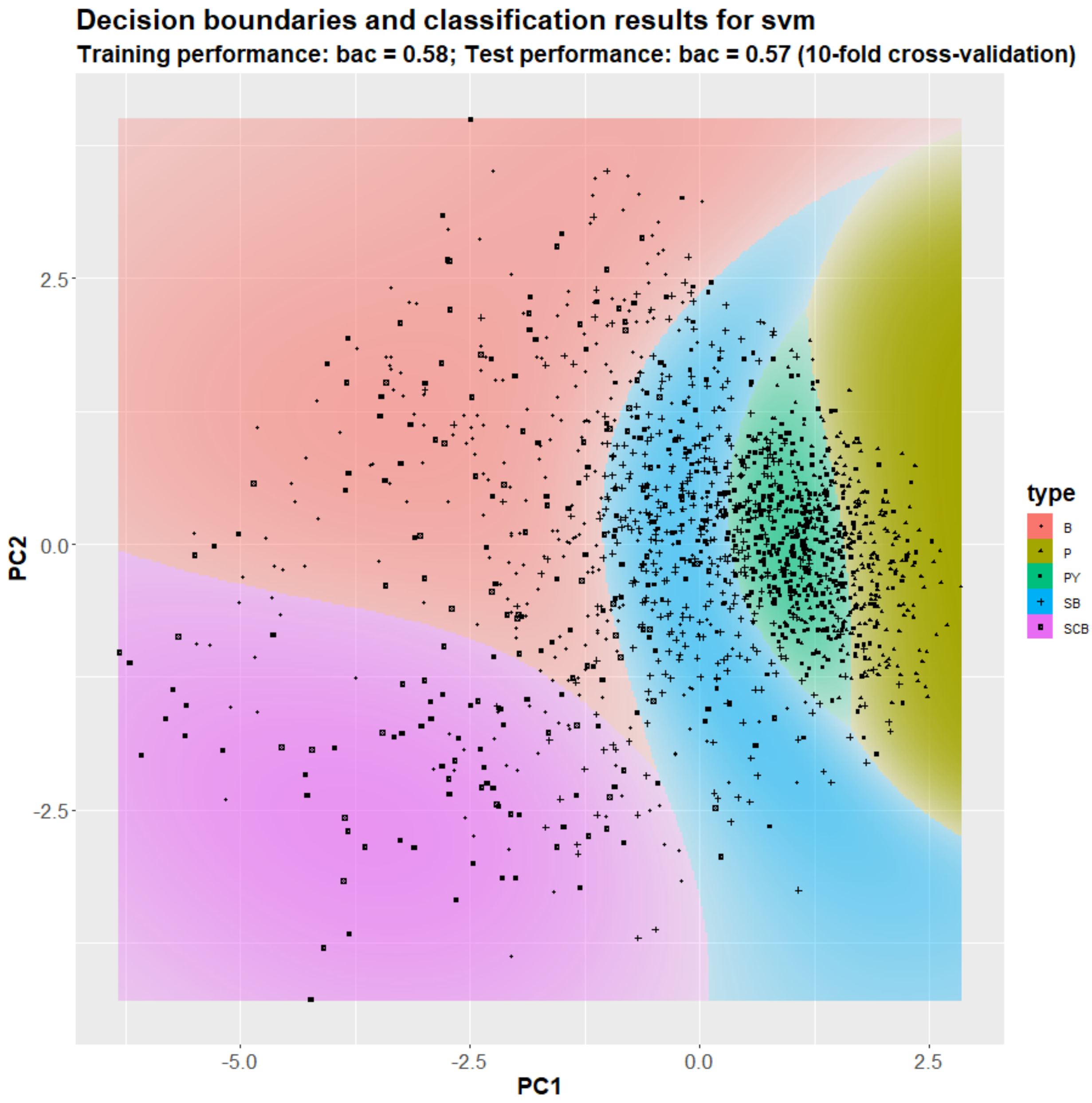

Figure 4. Decision boundaries and classification results for svm. Darker colors for the decision areas correspond to higher confidence in the prediction.

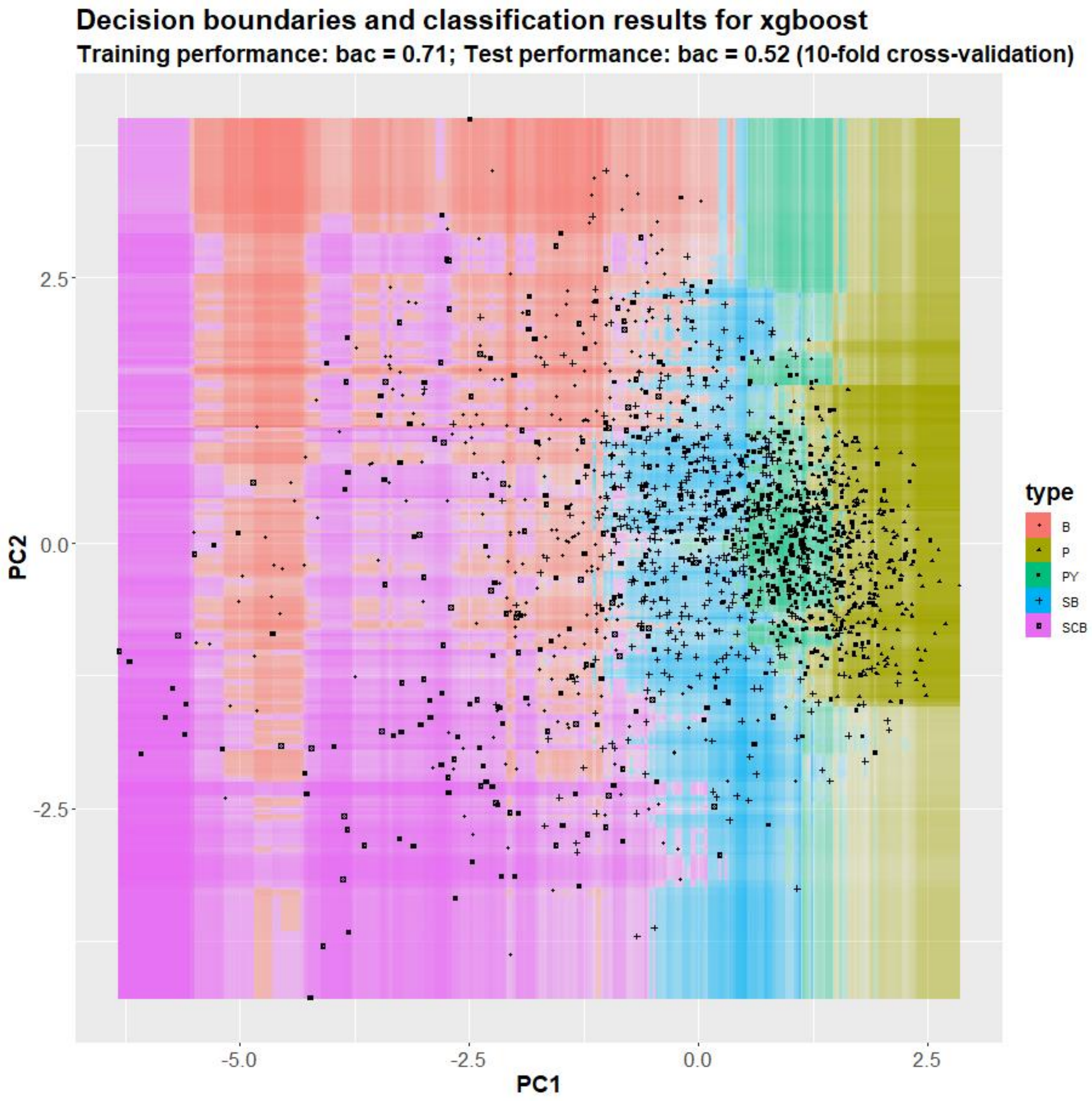

Figure 5. Decision boundaries and classification results for xgboost. Darker colors for the decision areas correspond to higher confidence in the prediction.
