# Supplementary material for: Improving the workflow to crack Small, Unbalanced, Noisy, but Genuine (SUNG) datasets in bioacoustics: The case of bonobo calls
Source: PLoS Comput Biol. 2023 Apr 13;19(4):e1010325. doi: 10.1371/journal.pcbi.1010325 (PMC10129004; doi:10.1371/journal.pcbi.1010325)
Supplement: Attachment — Submitted filename: Response to editor and reviewers.docx [file pcbi.1010325.s009.docx]

# Response to the editor and reviewers

Dear editor, dear reviewers,

We would first like to thank you for your insightful comments, requests for clarification, and suggestions. In this revised version of the manuscript, we have addressed all your comments and followed most of your suggestions.

The major modifications consist in:

- The clarification of the epistemological and methodological aspects that are generic to SUNG datasets, or conversely more specific to our case study on bonobos.
- The integration of UMAP (now in its supervised implementation S-UMAP) earlier in the workflow, leading to a straightforward explanation of its role and, more generally, a streamlined workflow.
- The addition and/or clarification of the essential methodological information throughout the paper to improve readability without excessive back-and-forth references to the comprehensive methodological section.
- The elaboration of a more thorough general discussion.
- As a consequence, the paper structure is now modified to integrate those changes:
  - A subsection “What is a SUNG dataset?” has been added to Section I.
  - S-UMAP is now presented at the beginning of Section II and the initial Section IV (dedicated to UMAP) has been removed.

We hope that this revised version of the manuscript will meet your expectations. In the rest of this document, our responses are in blue. To simplify, the reviewers’ minor comments are in green when we agreed with them and accordingly modified the manuscript without need for additional elaboration.

Sincerely,

The authors.

**Detailed response to the reviewers**

**Reviewer #1:**

Overall, the manuscript provides a thorough overview of the very typical kind of bioacoustics datasets, where having the data "speak for itself" is not straightforward. I warmly welcome the methodological focus on this type of data, and especially the nuanced view put forward by the authors. Rather than claiming a single solution for all analyses, they outline a process, demonstrate it on a running example, and add the necessary caveats in their conclusions. Moreover, importantly, the whole manuscript and proposed methodology is supported by an extensive range of computational experiments, resulting in a complete, well-founded study. Pointing out the sometimes subtle and often ignored issue of data leakage is a very welcome contribution to the methodology of the field.

### We thank reviewer #1 for this very positive assessment, and we are especially grateful for his pointing out the importance of the data leakage issue. We also much appreciated their thorough review and numerous suggestions aiming at improving the manuscript relevance and insight. We hope that our revision will meet their expectations.

Overall, I have 3 major points I would like to see addressed in a revision:

1) Whereas the writing is clear to read, the overall structure and transitions between different sections and paragraphs could be improved.

* Most importantly, parts of the experimental setup described in Section VI are necessary to clearly understand the results, but are not forward-referenced. As an example, the number of repetitions averaged out and contributing to the uncertainty estimates of the classification techniques (pDFA and ML) are not clearly introduced in Section II and "suddenly" turn up. (See detailed notes for more examples, in context.)

**Forward-referencing has now been extended and complex or uncommon notions have been introduced more carefully throughout the manuscript. The bulk of the technical details is still developed in the Methodology section but we hope that lisibility has improved.**

* Especially in the first section/half of the manuscript, it should be made sure that it si more clear which statements deal with the general properties of SUNG datasets, and which ones are describing the specifics of the bonobo dataset. The idea of illustrating the abstract properties of SUNG datasets with a concrete example is great, but the separation of these two should at all points be clear to the reader.

**Section I (“The bonobo recordings: a SUNG dataset”) now begins with a subsection named “What is a SUNG dataset?” aimed at clarifying the distinction between the general statements and the bonobo-specific aspects. This generic vs specific dimension is also mentioned in the description of Figure 1 and when necessary in other sections.**

* Some paragraphs have rather abrupt transitions, and obscure the overall line of reasoning (some examples noted further down).

**Agreed. We have consequently either reorganized sentences or added transitions when necessary.**

=> In general, the manuscript feels as if it has been heavily restructured before. It would strongly benefit from a thorough front-to-back reading by the authors, keeping in mind the reader's knowledge and perspective up to that point, adding the necessary forward and backward references, and introducing a more details, but still intuitive, description of the experiments before the results.

**For full disclosure, the manuscript had not been previously restructured (the detailed methodology was meant to be at the end) but we admit that we were not careful enough in introducing the notions and techniques beforehand. We hope that this revised version will meet the reviewer's expectations.**

2) The fourth and final part, visualising the data with UMAP and calculating silhouette scores, is not clearly reproducible and does not serve a valid purpose, as far as I can tell.

### We admit that the way we presented the UMAP approach was awkward and not sufficiently integrated to the main workflow. We thank the reviewer for driving our attention to this weakness and we have now adopted a much straightforward pipeline. UMAP is now directly incorporated at the beginning of the workflow in its supervised version (S-UMAP). Our rationale is that S-UMAP can be insightful for bioacousticians to understand a dataset structure, beyond its “discovery” power. It offers an elegant way to probe the consistency of the labels (call types and individuals) determined by the human experimenters against a graphical representation of the bonobo acoustic space. Moreover, S-UMAP silhouettes give a cue on the difficulty of the automatic classification task. We also argue that S-UMAP can additionally be useful to evaluate classifiers themselves or to identify potentially mislabeled calls, but for the sake of brevity and clarity, those aspects are only mentioned in the discussion. Hence, there is no more a specific (and admittingly inelegant) section devoted to UMAP.

* Firstly, the purpose: while I am 100% convinced visualisation of datasets is crucial, I cannot find the goal of this extra analysis step *at the end* of the pipeline. The whole manuscript presents a very typical supervised machine learning methodology, having a statistical algorithm learn the human-assigned "ground truth" based on each data point's extracted features. So the extra, unsupervised clustering step at the end to find categories seems useless. I will assume that the authors did have a good reason, and would like to hear more about this, but in the current form, the article does not motivate this aproach.

### Agreed. Insisting on the exploratory power of UMAP was misleading. In the new version we insist more on the interest of supervised UMAP as a visualization of the “ground truth” and as a way to check its consistency. We hope that the (S-)UMAP relevance is now more straightforward.

* Please note that the referenced article by Sainsburg et al., where UMAP is being used, has an entirely different purpose (i.e., exploring call repertoires and finding call categories). On the other hand, this manuscript's methodology before section IV presupposes existing categories, determined beforehand. The additional benefit of UMAP (or other unsupervised/clustering methods) is lost to me, *especially* at the end of the analysis pipeline. A short discussion of the high-level differences in purpose and approach with Sainsburg et al. could clarify.

**We hope that in the introduction, the purpose of using UMAP is now made clear and distinct from the “discovery” approach emphasized by Sainsburg and colleagues.**

* It is also not clear how, in the 2nd and 3rd application of UMAP, the predictions are "included". Neither in section IV, neither in the details of section VI could I distill the exact details that I need to know to reproduce this approach (potentially, page 36 describes this partly, but it should be made much clearer).

**Since S-UMAP is now applied to the raw features (and no more to the embeddings derived from the classifiers), the methodology is simpler. Initially, too many aspects were probably only accessible by reading the R code (accessible from the RMarkdown files).**

* Finally, it seems obvious that including the predictions (which previous results showed were 50 to 80% accurate) would improve the clustering. Since the classes are reasonably well predicted by the supervised models, and these classes are discrete values, an increased quality clustering seems very unsurprising. Moreover, the value of that clustering seems to not add any insights about the data, as the clustering of well-predicted classes of the input data conforming to the same input data's classes is a rather circular result? It is however hard to judge as I cannot reconstruct the exact details of how these predictions were included in the UMAP clustering.

**These UMAP embeddings derived from the classifiers have been removed.**

=> In general, the UMAP/visualiation section needs a big rework, clearly stating the intended purpose as well as how the input to the UMAP algorithm was constructed. In the current form, this section completely fails to convince me on both fronts, even though I would like to be and am generally hugely in favor of such visualisations approaches to better understand the structure of a dataset. This is an important issue to be resolved in order for this paper to be accepted and published.

**UMAP visualization is now streamlined in the workflow described in the paper and we hope that its relevance appears more clearly. As bioacousticians and/or data scientists, all of the authors consider that their potential is important to underline, for discovery but also for diagnostic, evaluation, and confirmation purposes.**

3) As far as I can tell, XGBoost is a software libary, providing generic gradient boosting implementations which can be used in many different context. Based on the authors' description as well as XGBoost's online documentation (https://xgboost.readthedocs.io/), the actual algorithm/supervised models trained are "gradient boosted trees". Please dig into this, figure out the correct terminology, and correct the manuscript accordingly.

### The terminology is now corrected.

**Minor comments and examples:**

### We addressed most of the reviewer’s comments below (through rewording, adding developments or references) without need for any additional answer from us. Those comments are reported in green below and the manuscript version with “track changes” turned on highlights the modifications. In contrast, when we judged that additional information would be helpful, we answer under each reviewer's comment (reported in black then).

- p1, 22. "UMAPs": I believe "UMAP" as the name of the algorithm can just be used in singular here.

- p3, 2. "discriminate data samples": it could be useful to introduce the concept of "features" here, while describing the data points and training/test data.

- p3, 11-13. A short and/or intuitive way to describe the way pDFA mitigates this risk, and what remaining limitations it has, this would make this section a lot more useful. At the very least, a reference for the interested reader to find out more about the "its limitations" would be appreciated.

- p5. In general (not just Figure 1), the resolution of the images and figures is very low. I don't know if this is releated to PDF quality or the printer, in my case, but the authors should verify that the figures have a high enough resolution and sharpness.

### We agree that the quality of the figures embedded in the manuscript is far from perfect. It seems that they have been degraded during the online submission procedure, but links to high-resolution versions of each figure were automatically added on the top of them at the end of the pdf file. We hope that this issue will be fixed in the production process if the paper is accepted.

- p5, caption. "Workflow implemented to analyze ...": Is this the proposed general workflow, the illustrated workflow with the bonobo data, or the workflow applied for this paper to set up experiments. Please clarify (and as stated before, try and keep the "general" case clearly distinct from the "specific" illustration with bonobo data).

- p6, 23. "shown in Figure 2": Looking forward to Figure 2, it does not contain a clear list of the call types (only scattered and duplicate abbreviations).

- p6, 34-35. "this kind of individual signature depending on the type of call": unclear what the authors are actually referring to. Please clarify/rephrase.

- p6, 36 - p7, 1. Example of a very abrupt transition. Also an example of jumping back and forth between SUNG-general and bonobo-specific sentences. In general, Section I and pages 6 and 7 could be improved b. making the links clearer between statements about bonobos and how this *relates* to typical issues in SUNG data.

- p7, 8-15: Great argument on the issue with small datasets and their caveats, and how bioacoustics data differs in important ways from speech!

- p8, 6 (and everywhere else). "F0": I've been told by reviewers before to use f0 (with 0 in subscript, ideally) as notation, and this does seem to be the more common notation. This also avoids confusion with formants.

- p9. However interesting the "Dataset distribution" is as visualisation, I am having a hard time intuitively reading and interpreting these areas. I am wondering if this is the most intuitive visualisation. E.g., what about either a heatmap (similar to Table 1, but colored by number), or stacked bars (one bar per animal, multiple segments per call type). Such visualisations would more easily allow comparison between the size of e.g. Vifijo and Djanoa (I have absolutely no idea which of the two has the most number of calls, because of their uncomparable shapes).

### The figure is meant as providing a visual sketch of the SUNG corpus consisting of bonobo calls. The treemap graph we chose is meant to illustrate the unbalance in data distribution and not to provide an accurate metric evaluation of this imbalance. We nevertheless modified the legend to be more explicit on the fact that the precise dataset breakdown by individual and type is given in Table 1, as rightly underlined by the reviewer.

- p9. Out of curiosity: what types of calls are the calls by Jill in part C? Adding this might satisfy this reader's curiosity.

- p9, caption. "Some individuals are recorded": probably not too important, but is every individual always recorded either inside or outside (which is how the statement sounds)? Or are just some recordings done inside and some outside, with inidviduals both recorded inside and outside (as seems more logical, but not clear from the sentence)?

- p10. "A sketch of across-individual variability": "... in Bonono vocalizations"? Again, it is not entirely clear here whether this subsection is meant as a general technique/workflow or just as background for this specific dataset. An effort to increase clarity (here, but also in general in the first half of the article) would be good.

### We hope that the reorganization of Section I with the “What is a SUNG dataset?” subsection in introduction and the modifications made following this reviewer’s comment will make this generic vs specific aspect clearer.

- p10. A lot more "F0"s.

- p10, 11. "like deterministic chaos": Please use a better acoustic term (something with "non-harmonic vocalisations" or "turbulent air flow" or so?)

### Deterministic chaos is indeed a term coined in bioacoustics to describe such phenomena. We added two references:

### Riede, T., Owren, M. J., & Arcadi, A. C. (2004). Nonlinear acoustics in pant hoots of common chimpanzees (Pan troglodytes): Frequency jumps, subharmonics, biphonation, and deterministic chaos. American Journal of Primatology, 64(3), 277–291. <https://doi.org/10.1002/ajp.20078>

### Wilden, I., Herzel, H., Peters, G., & Tembrock, G. (1998). Subharmonics, biphonation, and deterministic chaos in mammal vocalization. Bioacoustics, 9(3), 171-196. <https://doi.org/10.1080/09524622.1998.9753394>

- p10, figure 3. "PRAAT" was written "Praat" before.

- p10, figure 3. "50% or 80%": so these are centered around the median? So 50% is the inter-quartile range?

### Right. Areas are defined by the 25th and 75th percentiles, and the 10th and 90th percentiles, respectively.

- p10, figure 3. When printed (at least, for me), the grey area is as good as invisible. I did not notice it until I read the caption.

- p11, 3-4. "while it is more central": "... more central in duration". "more central" sounds weird for these 2 unrelated dimensions.

- p11, figure 4. Again, the resolutin (or printing quality) is very low somehow, making the left plot almost useless. But even with better quality, it it still a very cluttered and overlapping plot, so any extra effort to reduce this is appreciated. However, the general idea is pretty good-looking!

### We improved legibility by keeping only one larger figure displaying all the relevant information.

- p11, figure 4, caption. "1/10th": does this mean 1 10th of the duration, 1 10th of the pitch range? Please shortly clarify. Or, if not important, leave out the 1/10th and just keep "miniature", which should be good enough for the figure.

- p11, 14. "MFCC": worth mentioning (and referencing) that MFCC originate and are often used in human speech processing.

- p12, 17. "derived from decision trees": How? Is there any way of intuitively specifying? The current phrasing poses more questions than it answers.

- p12, 17. "considered as one of the best methods". For what? Picking methods is always a tradeoff, depending on context etc. Please refrain from such big claims or put them better in context.

- p12, 23-27. Thank you for nicely pre-empting the obvious "but deep learning!" comments and explaining why it is not an option for SUNG datasets!

- p13, 1-2. "instead .... true/false positives and negatives": FP/FN/TP/TN are directly related to accuracy (being TP/(TP + FP)) and recall. So the claim that they would be better for imbalanced classes (implied by the part of the paragraph on p12) does not make sense to me.

- p13, 26. The main issue with MFCCs is not per se their number. I would argue that MFCCs (and cepstrums) in general are hard to intuitively interpret, independent of how many there are.

### We changed “too many features” in “their large number” because we didn’t intend any negative connotation here. Instead, we wanted to highlight that by design, information is spread over a large number of dimensions leading to almost meaningless “importance” assignment to each individual dimension.

- p13, 19-34. Better link up the explanations of the 3 ensembling methods to their description. Already a "respectively" would do wonders, but by restructuring a bit more, it will become a lot clearer to the reader.

- p13, 33-39. Related to the major comment from above, but xgboost might be very confusing for the reader, as xgboost is presented as a basic, non-ensembled ML method. Please make sure to clarify this situation and avoid any confusion between the two contexts that "xgboost" might cause.

- p14, 1-7. Before, good arguments were made about keeping models simple (e.g., no deep networks). However, here, through ensembling, the model becomes suddenly a lot more complex. It would be good to discuss the link between data size and overfitting here, and discuss if ensembling would risk overfitting (and if not, why not?).

### The main rationale for excluding deep nets is rather that they are not the most adapted to the issues at stake with SUNG datasets than because they would be too complex. However, the simplicity argument – articulated to limited differences in performance compared to xgboost – is what led us to recommend svm rather than xgboost in the end. Regarding overfitting, the risk is not higher for ensembled learners than for simple classifiers as long as the hyper-parameter tuning and the evaluation are made properly.

- p14, 9. What is the task? Please first introduce the goal (one sentence is fine) before diving into the results.

- p14, 11. What is the size of the training/test set split?

- p14, 22. "best performances ... with xgboost": however, in 2 of the 3 cases, SVMs outperform xgboost on the same feature set? At least, when I look at Figure 5. Please resolve.

- p14. When claiming MFCC is not working well, it is using way more than the typical number of filters. The typical number of MFCC coefficients I've seen being used is 13 (12 + energy), possibly + delta and deltadelta. These 39 features are still more than the 7 or 20 features of bioacoustics or DCT feature sets, but way less than the huge 192 used here. So, in order to convince me and other readers here that MFCCs are not appropriate, adding a feature set with a way smaller numbrer of coefficients would be good.

### This comment definitely raises interesting points on the best acoustic parameterization in a cepstral domain. The default choice of 12 parameters estimated on ~24ms sliding frames is inherited from research developed for audio recordings done at limited sampling frequencies (typically 8kHz or 16kHz) and suitable for the human voice frequencies analyzed in the Source-Filter theory. To which extent those parameters are the best for other primates is an open question as far as we know, but addressing it would lead us too far in the bonobo-specific territory for most readers and could be detrimental to the paper lisibility.

### However, we implemented three additional more orthodox models based on the central frame of each call (static features; plus delta features; plus delta-delta features) in order to compare their performances to the vector summarizing the whole call as implemented in our princeps experiment. The results are degraded and this analysis is given in the Supporting information.

- p14, 30. "automatically extracted": it is not clear how DCT is more automatically extracted than the bioacoustics dataset. Both rely on having manual annotations and Praat's automatic pitch detection?

- p14, 32. "all seven configurations": which seven configurations? This is not described anywhere before this point and comes out of nowhere. (example of unclear order, as mentioned above)

- p14, 35. "acc" "bac": are these abbreviations used? They get introduced but not really used in the paragraphs after.

- p15, 1. This also goes for the stacked learners, so I don't get the "for the non-stacked learners" addition.

- p15, figure 5. It is not clear where the error bars originate. What is being repeated? (After reading further, I see that 100 samples are being taken, but this is not at all clear at this point in the manuscript.)

- p15, figure 5. It would be nice to have the chance level indicated with e.g. a dashed vertical line, for reference.

- p16, Table 2. Merge "config #" and "combined config" columns to not waste space. Just "configuration" should be clear enough

- p16, Table 2. A lot of nubmer here. I wonder if there's a better way (e.g. lightly coloring as some sort of heatmap) to make this more easily readable. I get lost with so many numbers to compare.

- p16, figure 6. Why? What is the interpretation of this figure? What do we learn from it? Please discuss the visualisation's insights.

- p17, figure 7. In principle, one could theoretically link up the features of the two sets. E.g. DCT2 would probably be linked to both f0 slopes. Potentially, a correlation between the two sets would be insightful.

### While this comment opens interesting avenues, we decided that developing this aspect would hinder the overall lisibility, being too bonobo-specific. We added a sentence explaining that further analyses can be done to better understand the relationships between the feature spaces and we specifically added a comment on dct2 and duration.

- p17, 7. Significance is now mentioned way earlier than in Task 1. I would suggest to follow the same structure of pragraphs, as to have these two sections maximally comparable.

- p17, 8. "accuracies": balanced or unbalanced?

- p17, 8-9. What is the chance level?

- p18, 3. "positions" is a weird metaphor carying over from the plot. Is there a better way of putting this?

- p18. No discussion of DCT vs bioacoustics features? In this case, DCT doesn't seem to be quite as good, and this adds a caveat to the previous results of "DCT being almost as good as BA". So it is worth discussing this, just like in Task 1.

- p19, 1. "Adoption of strategies to mitigate it": Which are these?? Again, lots of detailed information was missing before, and this only gets clear after reading section VI. But really, the way imbalanced data is being dealt with cannot just be dropped here. Especially as it is one of the 4 letters of SUNG, the central theme of the manuscript. This needs to be much clearer and the structure and order of things deserves rethinking.

### This issue is hopefully solved by the more extensive forward-referencing throughout the paper.

- p19, 1-3. An insightful conclusion drawn from the observed results! Very important, in the case of small data to note this!

- p20, figure 10. again, what are the error bars?

- p20, 6-10. Nice and clear introduction of the issue of data leakage!

- p20, 14. It was until now not clear at all that anything was being resampled! Again, this needs to be made a lot more clear, throughout the manuscript.

- p21, 17. "ignoring the constraint of predefined size for both sets": unclear to what is actually meant. Please clarify.

- p22, 2. "100 runs reported in the previous section": ??? I didn't know anything about 100 runs, in the previous section. I explicitly searched through the manuscript, and no 100 runs is being mentioned in section II!

- p22, 17. "..."

- p23, 3-4. It is unfortunate that it is called the "Skewed strategy". In the ideal case, one would not have to clarify to the reader that this worst case should not be attempted as a strategy in any way.

- p23, 6. What is BALI? Is there a reference or link or anything?

- p23, 11-12. I would go futher and say that the caveat is relevant in most cases. But yes, probably *even more* in the case of SUNG.

- p23-25. As mentioned above, it is absolutely unclear what data *exactly* UMAP runs on. This whole section needs to be clarified.

**Since S-UMAP is now applied to the raw features (and no more to the embeddings derived from the classifiers), the methodology is simpler and UMAP has now been incorporated to the main processing pipeline..**

- p25, 4. "the average distance to data points ..." -> "the minimum average distance to data points ..."

**The definition of the silhouette scores has been completely revised to be more transparent (see section 2, A graphical assessment: Supervised UMAP and section 5, Supervised UMAP and silhouette scores)**

- p27, 8-10. Surely, some sort of theoretical analysis should be able to link the DCT features to the BA measures (e.g., curvedness vs difference in start vs. end slope)? If you want to make a case of more research using DCT, this would be a useful extra discussion to convince people why DCT features are not just abstract numbers.

- p28, 16-23. Most of this text goes for UNsupervised methods and exploring groupings/clusters of data points. Please clarify how the situation is different in this manuscript, as you have labeled data and supervised methods.

- p28, 33. "adopting SVM": but in section II, claims are made that xgboost is better?

- p29, 35. Praat version number, for reproduceability?

- p30, 1. Praat was also used for harmonicity?

- p30, 12. "DCTHarm": This is the first time this is being used. Everywhere before it is just "DCT". What's the difference?

- p30, 19. "the second coefficient is directly proportional to the linear slope": I'm not sure if this is true, as it is not a linear slope (but rather half a period of a sine/cosine wave). It will capture the slope, yes, but it won't be directly proportional to a linear slope.

### Agreed. We rephrased the explanations on DCT.

- p30, 32. "-peep-yelp)": typo

- p31, 1-4. Why? Please clarify why when wrong. Why was the one value "missing"? Did Praat detect it as "unvoiced"? And why reconstruct rather than just leave out the one data point? This whole paragraph causes a lot more questions than it solves.

### For this specific call, Praat indeed failed to estimate f0 in the middle of the segment, returning NaN. We could obviously just drop this call but instead, we decided to implement the described technique that is able to cope with a larger number of missing data, a situation that can be quite common in SUNG datasets. This is now more clearly explained in the manuscript.

- p32, 29. What train/test split %?

- p32, 31-32. I don't really see the importance of mentioning 25-thread parallelization to the current article, here. It should have no influence on the precision or other results, and kind of interrupts the other sentences.

- p35, 27. "glmnet"? Sounds like the name of an R library. But what is the full name of the model/algorithm?

- p35, 31. Is there anything on BALI for readers to check, or to help reproduce this research? Any sort of reference or other information? Otherwise, this is really hard to reproduce the results.

- p36, 16-24. This whole part is rather unclear. It is not clear whether the 100 iterations are used as separate datapoints or as features of one data point or ... Please rewrite and clarify, potentially with an example of the sort of data you end up before UMAP.

p37, 13-18. In general, I don't see the point here (where no time performance is reported either way) to specify the full computational hardware and threading. But if the authors insist, why not.

### We just wanted to make explicit that the R code provided can run on a personal computer equipped with a powerful configuration but that it doesn’t require any larger computation infrastructure such as a cluster.

# Reviewer #2:

The manuscript tackles a quite common issue when analysing animal communication and vocal repertoires: datasets constituted by a small amount of calls, produced by few individuals, recorded in suboptimal conditions. The authors relied on the analysis of information encoding in bonobo repertoire, improving our understanding, to propose a methodological, operational workflow that can be used by researchers dealing with similar assessments. The analyses conducted employed different kinds of feature extraction, state-of-the-art classification techniques, supported by a thorough literature review, adopting two evaluation tasks (the individual identity and the call type identification). The results support the conclusions the authors drew and the helpful recommendations they suggested. The authors indeed found how adopting multiple feature sets may be good practice for extracting non-redundant information, proposed a generalizable practical approach that can be helpful when dealing with similar problems and recommendations on how adequately treat small and unbalanced datasets where calls occur in sequences, which can be true for many animal species. Methodology is broadly and accurately described, datasets and analysis codes are available and deposited in an appropriate and accessible repository, allowing to reproduce the study. Concluding, the work is scientifically sound, well constructed and presented. However, the different feature extraction, classification techniques, and evaluation tasks, lead to a workflow which can be a little hard to follow: I recommend to indicate what methods, how many call types, individuals, and sets are used in which task (for instance modifying figure 1 illustrating the workflow accordingly; see comment to P14-L32).

**We thank reviewer #2 for this very positive assessment. We hope that the revised version allows a better understanding of the workflow and that lisibility has been improved in the process. Figure 1 has been modified accordingly with additional information on the datasets themselves.**

Below punctual comments.

### We addressed most of the reviewer’s comments below (through rewording, adding developments or references) without need for any additional answer from us. Those comments are reported in green below and the manuscript version with “track changes” turned on highlights the modifications. In contrast, when we judged that additional information would be helpful, we answer under each reviewer's comment (reported in black then).

ABSTRACT

L7 For clarity, I suggest to add why 'SUNG datasets offer a distorted vision of communication systems'

INTRODUCTION

P2

L6 I would rethink 'success stories'

LL7-9 This sentence needs to be supported by a reference

L13 Expensive in terms of?

L14 Are they really invaluable though?

LL23-26 Is there a reference that can support this statement?

L28 Is there a reference regarding the 'playback experiments' that can be cited?

LL29-34 Please, rephrase this sentence

P3

L13 Is the pDFA still the standard approach in bioacoustic analyses though?

LL25-34 Nice! See also the findings of Sainburg et al. 2020 (10.1371/journal.pcbi.1008228) for a comparison among those methods.

P4

L17-18 'more accurate' and 'more comprehensive understanding' compared to?

L20 Which are the 'difficulties inherent in raw naturalistic dataset'?

LL27-28 This will be probably stated afterwards but how did you carefully choose an adequate performance metrics? What makes a performance metrics adequate?

P6

L16 What do you mean by 'ubiquitous'?

L23 From figure 2 is difficult to understand which are the ten call types

L26-27 '...tonal contour (general shape and duration)': how is the general shape quantified?

P7

L5 maybe 'corpora' instead of 'corpus'?

L16-18 I am not sure I understood this sentence

L29 'whether an acoustic feature is relevant' in discriminating the emitter's identity?

L37 '20 adult' how many males and females?

P8

L3 'vocalizations.. double-checked by two other experimenters': did you measure the inter-raters reliability? If so, how?

### The check procedure was based on the consensus of the two experimenters who worked together. It’s now made explicit in the paper.

L3 I believe the correct reference is Boersma and Weenink

P10

L3 'the whole corpus' excluding grunts and screams?

L4 Which kind of smoothing has been employed?

L4 Maybe 'coarse' instead of 'gross'?

L12 How has the 'harmonicity' been measured?

P11

LL12-17 To increase the understandability, I would add which parameters the Bioacustic, MFCC, and DCT set consider, respectively.

### The parameter list being quite long, we decided to keep them (and their inclusion in each dataset) in appendix (Table A1) rather than to include them in the main text. We hope that the reference to the Appendix and the short description provided in the main text in this section will be informative enough.

P12

L1 Can you expand the extraction of acoustic features section? How are the tonal contour (previous page) and the acoustic roughness measured?

### We kept most of the methodological details in the dedicated section in order to keep reading fluid but the essential notions are now more carefully introduced and forward-referenced when needed.

P14

L21 So MFCC are used to identify call types but not the individual identity?

### MFCC, like the other feature sets, are used both for call type and individual signature identification.

LL27-28 Can you indicate some references here?

L32 Which are the 'seven configurations'? I got it when reading table 2 but there are a lot of analyses and different sets employed for the different tasks. The readers will be probably benefit from a clear explanation of what methods, call types, and sets are used in which cases (for instance modifying figure 1 illustrating the workflow accordingly could be enough).

P20

L3 'confirming the multidimensional and complex nature of the individual vocal signature' is more a discussion.

L7 which is the relationship between these vocal sequences and the call types indicated in the previous paragraphs?

P21

L8-12 The third strategy represents an interesting approach!

Fig 11: So, if I got it right, is the influence of data leakage measured when classifying individual signatures only (besides the subset of three-call sequences)?

### Indeed, we only investigated data leakage for the classification of individual signatures (for the sake of simplicity). We have made this more explicit with an additional sentence.

RESULTS

P21

LL19-20 Cool result!

P22

LL14-15 '259 sequences consist of only one call, 111 consist of 2 calls': calls refers to the number of calls or to the call types? Do the sequences include more than one single call type?

p23

LL1-3 Interesting result

L6 'Tools such as BALI': link this to the relative paragraph (an in-text citation like see paragraph 'Evaluating data leakage / BaLi' is enough)

p26

LL8-9 Average silhouette values of 0.9 and 0.96 for call types and individuals identity, respectively. With regards to figures 13 and 14 how are these values averaged and measured?

**Given the reorganization of our paper, especially with respect to UMAP, the use of Spearman's correlation was no longer relevant and has been removed.**

LL13-15 Indeed

P27

L1 This sentence needs a reference

LL1-2 Can you expand this?

L7 Did your set include the same call type used in Keenan et al. 2016?

L31 interesting

P29

L24 Maybe something like 'tackling' instead of 'grasping'?

P32

L22 Can you add a reference here?

LL27-28 What does 'some observations are inherently more difficult to classify than other' mean?

P34

shouldn't the whole paragraph 'Tuning procedure' report at least some references supporting the choices that have been made?

P36

L8 Why using 15 neighbors if the analyses either 10 individuals or 5 call types?

### The number of neighbors in UMAP refers to the number of observations (i.e. calls, here) that are considered when computing the projection. It is important for tuning the weighing between the local vs global scales adopted for the projection, but it is not related to the underlying number of “classes”. Please note that UMAP representations are now only present at the beginning of Section II, in order to simplify the workflow.

# Reviewer #3:

I have reviewed the paper by Arnaud et al (PCOMPBIOL-D-22-00956).

I read the paper from Arnaud et al with great interest and applaud the authors for attempting to improve and accelerate the methodological tools available for the analysis and classification of animal vocalisations.

In principle I think this paper falls nicely within the remit of PLoS computational biology. I do still have a few suggestions and questions that it would be great to get feedback from the authors on.

**We thank reviewer #3 for this very positive assessment. We hope that the revised version addresses the issues raised below and especially that the new organization that more closely integrates UMAP in a streamlined workflow will meet her expectations.**

1. My biggest issue is that I struggled a little to see the bigger picture with this paper; specifically, what precisely are the authors interested in achieving with their analysis of bonobo vocalisations? From what I can tell the goals range from classifying call types, assessing the influence of sample size and noise on such classification, comparing different methods/approaches to assessing the shortcomings of previous core approaches in the field (i.e. pDFA) and investigating biological questions associated with the encoding of information sets in animal vocalisations. The reason I bring this up is because I worry that by trying to address so much in one single paper the authors risk readers being overwhelmed and the core message and advances of the paper being lost. If possible, I would therefore propose that the authors try to condense their goals perhaps to the ones that are most pressing and most relevant for the field at the moment. I would argue that even a comparison of different methods to resolve the different call types (i.e. repertoires) would already be incredibly informative and ultimately easier to digest for the field. Undertaking this with SUNG datasets adds the additional novel component that is so relevant to many researchers working with animal communication and really elevates the paper and its contribution. Highlighting the shortcomings of other approaches and attempting to capture additional variation as a result of phenotypic differences, I think, could be addressed in a follow up study or eluded to in the discussion as necessary future work.

### We thank reviewer #3 for this thorough explanation that echoes some of reviewer #1’s comments and our own reflections when preparing the initial manuscript. We aimed to provide a clear picture and a workflow that would help other researchers in bioacoustics to get a grasp on how to improve SUNG dataset processing. We considered that showing the process performed on a real challenging dataset that also presented an ethological interest per se (the bonobo dataset) was the most insightful way of proceeding. We thus cut a balance between giving not sufficient information on the vocal communication system itself to emphasize genericity and producing a much more thorough – but more specific – analysis of the results on the bonobo dataset. This comment nevertheless suggests that this balance could be improved, or at least that our main goals could be made clearer and the manuscript structure simplified.

### In this revised version, we decided to keep the information provided in the initial manuscript because they highlight important aspects of SUNG datasets, but we reworked both Introduction and Section I (adding a subsection “What is a SUNG dataset?”). Additionally, the UMAP visualization is now integrated in the main workflow and no longer as an additional approach, which should make the paper more streamlined. We hope that reviewer #3 will agree with us that these changes make the paper valuable and clear enough.

2. I think a clearer reasoning or justification for using bonobo vocal data would also be valuable. Perhaps mention that, of the primates, great ape vocal data is notoriously difficult to collect and is riddled with issues resulting from sample size and background noise etc. Monkey and non-primate data sets suffer from these issues less because there are often more groups from which data can be collected whereas with apes, researchers are often restricted to working with one group leading to limited subjects and ultimately constrained sub-optimal data sets.

### Agreed. This aspect is now mentioned at the end of subsection “What is a SUNG dataset?” in Section I.

3. I think it would be useful to provide spectrograms of the main 5 call types the researchers focus on for their analyses.

### Done (Figure A1 in Appendix).

4. Since the bonobo repertoire is rather tonal in its nature, as far as I can see, the authors are not confronted with comparing voiced and unvoiced calls. Since this is not always the case in many animal call repertoires, some guidance with regards to the analysis of vocal repertoires comprising both voiced and unvoiced calls would be useful. In my experience the absence of an F0 can massively complicate call classification and comparisons and a suitable solution (other than relying on less comprehensive non-F0 based measures or features) is not available.

### This aspect is now mentioned in the general discussion (Section Main findings>Features describing acoustic signals).

Have the authors made all data and (if applicable) computational code underlying the findings in their manuscript fully available?

The [PLOS Data policy](https://journals.plos.org/ploscompbiol/s/materials-and-software-sharing) requires authors to make all data and code underlying the findings described in their manuscript fully available without restriction, with rare exception (please refer to the Data Availability Statement in the manuscript PDF file). The data and code should be provided as part of the manuscript or its supporting information, or deposited to a public repository. For example, in addition to summary statistics, the data points behind means, medians and variance measures should be available. If there are restrictions on publicly sharing data or code —e.g. participant privacy or use of data from a third party—those must be specified.

Reviewer #1: No: The data has been made available (and is the most important part), but no code has been attached.

### The code was and is accessible in the html files available in the Github repository mentioned in the article. (<https://github.com/keruiduo/SupplMatBonobos>). The point is to embed the relevant code with each section of the results.

However, I leave this up to the editor on whether this is necessary. If the right methods and version numbers are added, this would also be fine with me.

Reviewer #2: Yes

Reviewer #3: Yes

PLOS authors have the option to publish the peer review history of their article ([what does this mean?](https://journals.plos.org/ploscompbiol/s/editorial-and-peer-review-process#loc-peer-review-history)). If published, this will include your full peer review and any attached files.

If you choose “no”, your identity will remain anonymous but your review may still be made public.

Do you want your identity to be public for this peer review? For information about this choice, including consent withdrawal, please see our [Privacy Policy](https://www.plos.org/privacy-policy).

Reviewer #1: No

Reviewer #2: No

Reviewer #3: No

Figure Files:

While revising your submission, please upload your figure files to the Preflight Analysis and Conversion Engine (PACE) digital diagnostic tool, [https://pacev2.apexcovantage.com](https://pacev2.apexcovantage.com/). PACE helps ensure that figures meet PLOS requirements. To use PACE, you must first register as a user. Then, login and navigate to the UPLOAD tab, where you will find detailed instructions on how to use the tool. If you encounter any issues or have any questions when using PACE, please email us at figures@plos.org.

Data Requirements:

Please note that, as a condition of publication, PLOS' data policy requires that you make available all data used to draw the conclusions outlined in your manuscript. Data must be deposited in an appropriate repository, included within the body of the manuscript, or uploaded as supporting information. This includes all numerical values that were used to generate graphs, histograms etc.. For an example in PLOS Biology see here: http://www.plosbiology.org/article/info%3Adoi%2F10.1371%2Fjournal.pbio.1001908#s5.

Reproducibility:

To enhance the reproducibility of your results, we recommend that you deposit your laboratory protocols in protocols.io, where a protocol can be assigned its own identifier (DOI) such that it can be cited independently in the future. Additionally, PLOS ONE offers an option to publish peer-reviewed clinical study protocols. Read more information on sharing protocols at<https://plos.org/protocols?utm_medium=editorial-email&utm_source=authorletters&utm_campaign=protocols>

In compliance with data protection regulations, you may request that we remove your personal registration details at any time. [(Remove my information/details)](https://www.editorialmanager.com/pcompbiol/login.asp?a=r). Please contact the publication office if you have any questions.
