# Supplementary material for: Improving the workflow to crack Small, Unbalanced, Noisy, but Genuine (SUNG) datasets in bioacoustics: The case of bonobo calls
Source: PLoS Comput Biol. 2023 Apr 13;19(4):e1010325. doi: 10.1371/journal.pcbi.1010325 (PMC10129004; doi:10.1371/journal.pcbi.1010325)
Supplement: Attachment — Submitted filename: 2nd response to editor.docx [file pcbi.1010325.s010.docx]

# Second response to the editor

Dear editor,

Thank you for your comments on the first revised version of our paper. As already discussed with you earlier, we have tried of course to tackle each of them the best we could – please find some precisions below.

We hope that this new version of the manuscript will meet your expectations. Please kindly let us know.

Sincerely,

The authors.

**Detailed answer to the comments and suggestions**

1. I also appreciate the very significant contribution of the permuted-DFA, but in your introduction it sounds like it is a concept/approach that was originally developed by Mundry and Sommer. Their contribution was essential in bioacoustics but the concepts of non-independence of data (think random effects in mixed-effect modeling), permutation tests and cross-validation were well described before that in the statistical literature (and prior to the data leakage formulation used in machine learning – checkout the wikipedia page for permutation test for example – it will bring you to the 1930…). I would introduce the pDFA (and the contribution of Mundry and Sommer to its application in bioacoustics) with that more inclusive historical/statistical perspective.

- **We have added some references and modified the text to i) better state that permutation tests are nothing new and can be traced back in particular to Fischer, ii) be more explicit as to Mundry and Sommer’s contribution.**

2. Along the same lines, in the rest of the paper, you keep on using pDFA but in fact, all your methods use a form on permutation test by performing the right type of cross validation (see below). The difference between the classifiers are a linear discriminant analysis (DFA) versus non-linear methods that find other boundaries between classes (including potentially multimodal/donuts in case of xgboost). Thus, first, I would suggest – using DFA vs other classifiers instead of pDFA vs other classifiers in the rest of the manuscript. Second, in your discussion, you mention things like “Because it may not be sensitive enough, pDFA…” ( p. 32) when you compare the DFA to other classifiers Saying “not sensitive enough” is quite vague. I encourage you to do better and explain the differences between classifiers both more explicitly and more didactically. For example, I suggest you try to generate a figure with the different decision boundaries that are obtained with the different classifiers.

- **We totally agree with you that all our classification approaches rely on permutation tests to establish performance baselines. We have replaced pDFA with DFA whenever this made sense, and merged two partly redundant paragraphs where explanations on permutation were given – one in ‘Automatic classification approaches and evaluation methodology’ and the other in ‘Detailed methodology / DFA’ – into a single one. Hopefully things are clearer now, and readers will readily understand we have a unified approach to performance baselines.**
- **We have modified the sentence p. 32 you refer to and added a new document in Supplementary Information where we illustrate the different decision boundaries obtained with DFA, svm and xgboost.**

3. Figure 1 A. I know that this is a workflow figure but the cartoon for the spectro-temporal templates is a bit cryptic. I know it becomes clear as one reads the paper but why not add x and y axis labels? .Also note that you illustrate with only 4 templates for call types (and not 10) and in the individual plot maybe you should have an individual grouping color (otherwise it looks like more than 20 individuals)?

- **We have redrawn, updated and simplified Figure 1. Labels have been added.**

4. P. 10 “Vocalizations were manually segmented, identified and then double-checked by two other experimenters (through a consensus decision-making) with Praat (Boersma & Weenink, 2022), based on the visual inspection of signals on spectrograms.” This sentence is hard to parse - I suggest “Vocalizations were manually segmented, identified and then double-checked by two other experimenters (through a consensus decision-making) based on a visual inspection of the signals on spectrograms and an estimation of the fundamental frequency, fo, using the speech analysis software Praat (citation).” This will also help with the sentence that starts with “The audio quality of the recordings was variable…” where you mention fo and Praat again without defining Fo.

- **This is better indeed. We have rewritten the sentence according to your suggestion.**

5. P. 18. Define AUC once as the Area Under the ROC Curve.

- **Done**

6. Fig. 7 The font size in this confusion matrix is too small. Use all the space you have. This comment is valid for all your figures. Please take a good look at your font sizes in all the figures and make then legible and more appealing.

- **We have prepared new figures with increased font size (Figures 7, 8, 10 and 11). Textual elements should now be easier to read – it is true they were way too small before.**

7. There is no specific reason that prevents you from using DFA with a high dimensional feature spaces any more so that with other classifiers. In fact, one could argue that it is the opposite. DFA is a simpler model than SVN, NN, or tree classifiers in the sense that there are fewer parameters to fit per feature dimension. Just as there are regularization parameters (hyperparameters) for the other classifiers, you could regularize the DFA to prevent overfitting – for example by performing a PCA on your feature space and preserving a variable number of PCs. This could be used on the MFCC feature space for example (see Elie and Theunissen, 2016). A regularized DFA might also boost your performance for the other feature spaces.

- **We have relied on a PCA to consider the DFA – MFCC configuration. Details about the approach followed, along with a figure to explain how we selected the number of principal components (Figure 15), are reported in the paper.**
- **Figures 6 and 9 and Tables 2 and 3 have been revised to show the new results for DFA – MFCC.**

8. In your description of the permuted DFA (p.37), it was unclear to me whether the permutation was performed within each group along the dimension not being tested or across all groups. For example, if you are testing for call-type, one should preserve the identity information and permute the call-type label within each individual but not across all individuals. And vice-versa for testing for identity (Note this is what was done in Elie and Theunissen, 2016 for call types – read cross-validation section on p. 294 - and Elie and Theunissen, 2018 for caller id). Did you do this? If so, please be more explicit about it in the methods. If not, I suggest that you perform the permutation using this more rigorous approach. It deals with the unbalanced data (or, equivalently, with the random effect of id when one analyzes call-type).

- **As we did not initially follow the more conservative approach you suggest for the estimation of our baselines, we have implemented it and explained it in the paper. Figures 6 and 9 have been updated to show the new baselines.**

9. Related to point 7 but for generating a fair testing set: I appreciate the fact that you use Bali to generate testing sets with specific objectives that control for random effects (such as two sounds coming from the same sequence). Just as in the permuted DFA, a strong approach is to generate a testing set that deals with the lack of independence in individual samples (also known as a random effect creating data leakage). For call-type, the testing sets could only have individuals that are not in training set. For id, the testing set could have call types that are not in the training set (or you should perform an analysis per call type). For the sequence effect, the sequences need to be all in the training set or all in the testing set, etc. If you do this, you will clearly get the most conservative estimate. And I am not completely sure what one would gain by applying the genetic algorithm. As far as I can tell you need a genetic algorithm because of you are getting the best solution for a fixed sized testing set (please correct me if I don’t understand – and if so make sure to also state more explicitly the reason for using Bali in the manuscript). Thus, you might have to generate testing sets of different sizes (for example as you include and exclude different individuals for assessing call type discrimination). But I don’t think this is a problem – you can always to the correct weighting to get the average performance.

- **We have expanded the section of the paper on leakage with i) an introduction on the issue and ii) a third part introducing and discussing the approach you depict. We have implemented it with a reduced dataset, and the exact results along with some considerations can be found in a new document in Supplementary Information. A new figure was created to illustrate our precise approach (Figure 14).**
- **We argue that the situation is quite tricky, especially given the SUNG nature of our dataset. Different approaches/options could in fact be considered, and the whole issue would likely require a lot of additional work which would take us a bit too far from the core of our study.**

**Please note that we had to slightly modify the structure of the article since the Editorial Office requested that we have a ‘Results’ section.**
